# 3D Point-of-Intention Determination Using a Multimodal Fusion of Hand Pointing and Eye Gaze for a 3D Display

**DOI:** 10.3390/s21041155

**Published:** 2021-02-06

**Authors:** Suparat Yeamkuan, Kosin Chamnongthai

**Affiliations:** Department of Electronic and Telecommunication Engineering, Faculty of Engineering, King Mongkut’s University of Technology Thonburi, Bangkok 10140, Thailand; suparat.y@mail.kmutt.ac.th

**Keywords:** multimodal systems, sensor fusion, eye tracking, hand tracking, hand recognition

## Abstract

This paper proposes a three-dimensional (3D) point-of-intention (POI) determination method using multimodal fusion between hand pointing and eye gaze for a 3D virtual display. In the method, the finger joint forms of the pointing hand sensed by a Leap Motion sensor are first detected as pointing intention candidates. Subsequently, differences with neighboring frames, which should be during hand pointing period, are checked by AND logic with the hand-pointing intention candidates. A crossing point between the eye gaze and hand pointing lines is finally decided by the closest distance concept. In order to evaluate the performance of the proposed method, experiments with ten participants, in which they looked at and pointed at nine test points for approximately five second each, were performed. The experimental results show the proposed method measures 3D POIs at 75 cm, 85 cm, and 95 cm with average distance errors of 4.67%, 5.38%, and 5.71%, respectively.

## 1. Introduction

Basically, intention is the philosophical ability of the mind to form representations, and it is a logical concept for doing something with a goal [1]. Humans communicate intention to others for informing what they desire based on their decision making. If a machine can detect human intention, it may be able to serve the human properly and safely. For instance, elderly individuals and patients feel it convenient to command against facilities in smart homes [2]. Collaborative robots might work safer with human workers in factories [3,4]. Medical facilities in hospitals would become more intelligent in assisting medical doctors to cure patients [5,6]. Vehicles would be prevented safety in the case that drivers do not concentrate on driving, and facilities in driverless vehicles serve passengers in being more friendly and convenient. Therefore, human intention detection is regarded as a key function for the development of intelligent machines. In fact, it is difficult to understand human intention, which is originally kept in the mind. Although humans express their intention in some ways, such as speech, touch, gestures, and so on, to communicate to others, it is difficult for machines that are based on current technologies to detect and understand the human intention. Intention detection can be functionally analyzed into a couple of functions that are mode switching between intention and non-intention and intention target detection.

In order to implement the required functions, approaches that are based on touch, speech, and gestures have recently been proposed and developed. Currently, the touch approach is popularly used in mobile devices, which are used in cases of short distances between humans and machines [7]. For instance, touch screen-based interactions of car infotainment [8], smart home operation panels [9,10], etc. Although methods in the touch approach are not smooth and friendly as speech communication between human and human, the methods are available and reliable in limited distance. As methods available for farther distance, the speech approach seems to work similarly to natural human-to-human commands and it may be preferred by users. For instance, speech activation for the control of home’s equipment [11,12], speech-based control for music and mobile phone infotainment [13], etc. However, it is not robust against noise, including signal noise and the conversations of other users. On the other hand, the gesture approach, which is suitable for remote control and seems natural for human commands, may be very effective for applications in human environments and vehicles [14]. Moreover, elderly persons, who are inconvenient for movement, and disabled persons, such as those with hearing impairments, who usually use sign language in daily life, may be able to use gestures to conveniently control devices in their living environments and inside the vehicle in the same way as persons without such disabilities.

Future facilities in vehicles and environments of humans, such as homes, working offices, hospitals, production factories, and cars, tend to be smart and friendly for humans [15]. For instance, remote control using human gestures at a smart home, virtual switching for commanding robots in a smart office, hospital, and production factory, gesture-based navigation for a driverless car, etc. These functions basically work based on communication between humans and machines, called human-machine interfaces. Flexible and convenient interfaces using hand pointing and eye gazes as useful combined gestures for remote control are required for switching between intention and non-intention modes to facilitate communication between humans and machines in future vehicles and environments. Normally, humans perform their gestures, such as pointing and watching at an object called the intended point for intention expression on the object. In practice, intended points that are allocated on two-dimensional (2D) screens can be detected by pointing and gazing in an area of interest (AOI) on a virtual screen, and the AOI is simply classified by the area ratio on the virtual screen [16]. This may not be matched with an intended point in a three-dimensional (3D) space, since the AOI size is gradually changed based on the distance from eyes to the object.

The authors of this paper assume that a target point-of-intention (POI) is allocated anywhere in a 3D space and aims to develop a practical intention estimation system while using a multimodal fusion of hand pointing and eye gaze. According to a study of psychologists that human eyes usually saccade within approximately 5–10 degrees for finding a fixation on an object [17,18], our paper mainly contributes to propose a multimodal fusion between hand pointing and eye gaze for 3D POI detection. In practice, the intention has to be determined first, and then the intended point would be estimated. In order to determine the intention, the hand pointing vector, which is the most important gesture to express intention is ideally assumed to cross with the eye gaze vector at an intended point in a 3D space. In reality, some errors may occur due to human eye saccades, and it is hard to find the crossing point. Therefore, this paper sets a space of interest (SOI) representing the vector of eye gaze based on the psychologist knowledge to find a crossing point with the hand pointing vector for intention determination, and contributes multimodal fusion method of hand pointing and eye gaze. Moreover, a hand that is freely moved needs to be determined as hand pointing when users intend to point to a 3D intended point. This paper also proposes a method of hand pointing determination while using projected 2D templates of hand pointing and straight index finger detection.

This paper is organized, as follows: Section 2 describes related works. Section 3 and Section 4 provide an analysis of the problem, an analysis of the 3D POI, and the system overview. The details of the proposed method and the corresponding experiments and results are reported in Section 5 and Section 6, respectively. Section 7 presents a discussion. Finally, the paper is concluded in Section 8.

## 2. Related Work

In human POI detection, while hand pointing has been confirmed as the most important gesture for intention communication [19], many researchers have aimed to develop intention detection systems that are based on hand pointing.

Generally, hand pointing is psychologically accepted to express the pointing intention and pointing direction of humans, especially for remote distances. Some researchers have chosen this approach. For instance, Q. De Smedt et al. [20] presented skeleton-based hand gesture recognition in a dynamic system and then compared it with methods utilizing depth information. The results showed that the overall accuracy of skeleton-based methods was better than that of depth-based methods. B. I. Ahmad et al. [21] proposed a hand pointing gesture with a Leap Motion sensor as the hand tracker to determine items that were selected by a user on a 2D screen. The method worked well for 2D displays. B. Daniel [22] presented an evaluation of Leap Motion as a contact-free hand pointing for gesture-based human machine interaction. The method using hand and fingertip positions is detected in coordinates relative to the center of the controller in coordinate system. The results show good accuracy declared at the sub-millimeter scale based on the analysis by Fitts’ law. S. S. Das [23] proposed a method of estimating precise pointing directions using depth data with a Kinect depth sensor. The method effectively estimated pointing directions. All of the mentioned related works used a vector of hand pointing for the determination of POI, so that the methods could deal excellently with POI on 2D screen. The crossing point between the hand pointing vector and the plane of a 2D screen was assumed to be the POI. However, these might not be appropriate for POI detection in a 3D space, since the detection of POI in a 3D space geometrically required the crossing point of at least a couple of vectors. Therefore, another vector should be fused with the hand pointing one, and multimodal fusion between hand pointing and others became challenging for the 3D-POI-detection system development.

Researchers of related works have fused hand gesture, speech, and eye gaze with hand pointing as a multimodal fusion [24] for POI detection on a 2D screen. The multimodal fusion of hand pointing and hand gesture [25] played roles of intention expression, such as pointer on the screen, click timing, rotation, and so on. The method used the natural behavior of humans as an advantage, but users have to learn command formats in advance. In the group of multimodal fusion of hand pointing and speech [26], the speech, which normally was regarded as friendly for users, was conveniently used as command, but the speech was sensitive with noise and crosstalk as the drawback. On the other hand, the methods [16,27,28,29] in the group of multimodal fusion between a hand pointing and eye gaze are considered to be a useful tool for elderly persons, patients, and disabled persons, and the crossing point between hand pointing and eye gaze vectors would be workable for POI detection, not only on a 2D screen, but also in a 3D space. Research works in the group of multimodal fusion between a hand pointing and eye gaze are as follows.

N. Chuan and A. Sivaji [16] presented a method for combining eye gaze and hand tracking for the pointer in a human-machine interface. In this method, the hand is tracked by the Kinect depth sensor in terms of data voxels, and the AOI of the eye gaze that is sensed by the eye tracker is then matched with the hand pointing direction on a 2D screen. P. Gowdham and B. Pradipta [27] presented a system utilizing the combination of eye gaze and finger tracking for a projected display in automotive and military aviation environments. The method worked well in applications for touch buttons on 2D screens. B. I. Ahmad et al. [28] performed experiments in real situations and confirmed the possibility of combining gestures and eye-gaze trackers for intention expression with 2D displays in vehicles. The results of the measurements were also reported. F. Roider and T. Gross [29] proposed the integration of eye gaze and pointing gestures while driving for a 2D virtual screen. The method was shown to be feasible for applications on 2D virtual screens.

To conclude, regarding the research and development of combinations of hand pointing and eye gaze, researchers have contributed to the tracking of human intention to touch a button at remote distances based on physical 2D screens as well as virtual 2D screens. Because the research was intended to solve problems for 2D screens, the touched button was ultimately estimated in a 2D space. These methods could be applied to problems of 2D screens by matching the hand pointing direction with the AOI of the eye gaze.

## 3. Analysis of 3D POI

**Figure 1:**
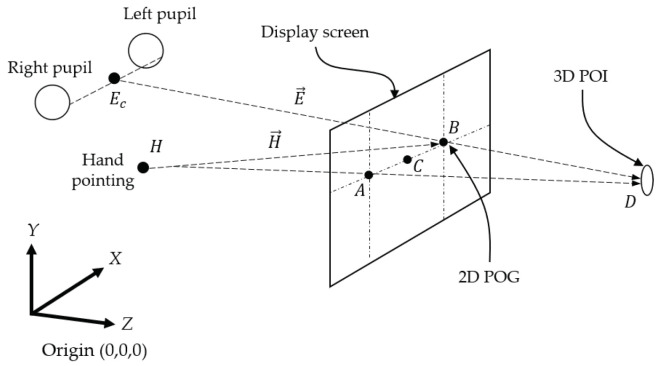
Three-dimensional point-of-intention (3D POI) determined through the eye gaze and hand pointing.

Suppose that there exist 3D coordinates (*X*,*Y*,*Z*) originating at an origin point (0, 0, 0), as shown in Figure 1, and a 3D POI is located at any position in the 3D coordinates. In conventional methods using the hand pointing and eye gaze [16,27,29], if the 3D POI is located on a display screen (*A*), both the vectors of hand pointing ($\vec{H}$) and eye gaze ($\vec{E}$) may ideally be combined at the 3D POI located at point *A* on the display screen. The position of the 3D POI on the display screen in this case is mathematically measured because the depth of the display screen is known. If the 3D POI is located somewhere in the 3D space and does not exist on the display screen, for example, both of the vectors of the hand pointing and eye gaze may point to point *D*, which is assumed to be the 3D POI. However, conventional methods determine the points of the hand pointing (*A*) and eye gaze (*B*) on the display screen, and then draw an AOI and find the middle point (*C*) at the closest distance as the POI.

Although errors occur according to these methods, they can be used in many applications that require users to choose areas on a display screen; however, they are not suitable for 3D virtual and real display screens in which the depths are not known. Recently, an excellent tool, called an eye tracker [30,31], emerged, which can detect the eye pupils and create a ray representing the eye gaze that is drawn from the middle point ($E_{c}$) between the pupils to a point on a fixed display screen. The display screen is studied for use as a virtual screen through calibration in advance, and the vector drawn from the middle point of both pupils passing through a point on the virtual screen has been shown to be able to be used as an eye gaze vector in 3D space [32]. Ref. [33] proposed translating the head by a short distance to find another eye gaze vector and using the crossing point of the two eye gaze vectors as the 3D POI. The constraint of the method is that users have to move their heads slightly. However, users are not allowed to move their heads, even slightly, in some environments, such as cockpits, for safety reasons. Therefore, a 3D POI detection method without head movement is also required. If the eye gaze is used as a vector passing through a 3D POI, and hand pointing, which is already considered to be a powerful action for intention expression [34,35], is fused with the eye gaze to find a crossing point, the position of the 3D POI can be geometrically obtained, as follows:

(1) $$D=E_{c}+\left(\vec{E}*\left(\frac{\left(H-E_{c}\right)\times \vec{H}}{\vec{E}\times \vec{H}}\right)\right)$$

where, *H* represents the tip of the index finger.

### 3.1. Fusion of Hand Pointing and Eye Gaze as Human Intention

**Figure 2:**
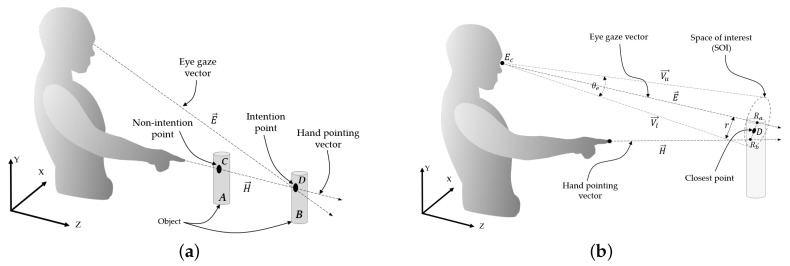
Human intention determined through the eye gaze and hand pointing: (**a**) Human intention through the eye gaze and hand pointing and (**b**) space of interest (SOI) and hand pointing during identification of intention.

Pointing in a single-hand gesture has been shown to be an effective way of human intention expression, according to [34,36]. When a single hand gestures by pointing to a point in 3D coordinates [37], it can be detected as an intention regarding all the points. If there is more than one candidate point on the pointing line (e.g., *C*, *D*, etc.), as shown in Figure 2a, it is difficult to determine the intended point. In this case, the human eye gaze assists in confirming the intended position, as introduced in [38]. Therefore, our paper proposes using the fusion of a pointing hand gesture and the eye gaze for intended point determination. A crossing point (*D* in Figure 2a) between the hand pointing line and eye gaze line, which represents a human intention, can be mathematically obtained from 3D coordinates in an ideal case, as shown in Equation (1). However, human eyes always perform saccades between fixation points [18], and sensor devices may not be able to address this in real situations, so it is quite difficult to correctly detect an eye gaze and then an intention point. Human line-of-sight rays may move within an approximate range of 5–10 degrees due to saccades, according to research works in psychological experiments [39,40]. Therefore, this paper sets the maximum cornea movement range covering saccades, which is called the SOI, as shown in Figure 2b.

In order to determine the SOI, while the light ray $\vec{E}$ making a right angle with the straight-line between both eyes and passing through a fixation point (*D*) is assumed to cross the hand pointing vector at the maximum distance, line-of-sight rays that are assumed to move utmost 10 degrees will saccade in a circle range. This circle range is exactly the SOI, and the radius (*r*) of the SOI can be obtained by the following equation:

(2) $$r=E_{Length}\times \tan\left(\frac{\theta_{e}}{2}\right)$$

where,

(3) $$E_{Length}=\sqrt{{\left(R_{a}\left(x\right)-E_{c}\left(x\right)\right)}^{2}+{\left(R_{a}\left(y\right)-E_{c}\left(y\right)\right)}^{2}+{\left(R_{a}\left(z\right)-E_{c}\left(z\right)\right)}^{2}}$$

The crossing point between the light ray $\vec{E}$ and hand pointing $\vec{H}$ should be in the SOI because the SOI is assumed to cover the maximum cornea movement range. In reality, both of the mentioned vectors sometimes approach close without crossing, and the closest point should be considered as the crossing point or intended point under the condition of SOI. Therefore, the closest point is guaranteed to be the intended point by the following equation:

(4) $$D_{Length}<r\Rightarrow Closest\;Point$$

where,

(5) $$D_{Length}=\sqrt{{\left(R_{a}\left(x\right)-R_{b}\left(x\right)\right)}^{2}+{\left(R_{a}\left(y\right)-R_{b}\left(y\right)\right)}^{2}+{\left(R_{a}\left(z\right)-R_{b}\left(z\right)\right)}^{2}}$$

In order to implement an automatic system, it is absolutely necessary to define the mode of intention. If we define the intention point by the crossing point between the pointing hand and eye gaze, as mentioned earlier, and the intention mode may be switched on by finding this crossing point. However, eye gaze errors usually occur due to saccades and sensor devices, and a crossing point that should be found may not be detected, even though a passenger is displaying an intention. This paper considers errors related to the saccades of both the left and right eyes because the human eye naturally has limitations of movement and sight within a range, called the visual field [41], as shown in Figure 2b. The visual field is enlarged as a cylinder surrounding the eye gaze vector, and its diameter is similar to the range between the two eyes, which is the SOI. In this paper, the occurrence of a close point between the SOI and a hand pointing line is considered to indicate a change in the intention mode.

### 3.2. Eye Gaze Vector

**Figure 3:**
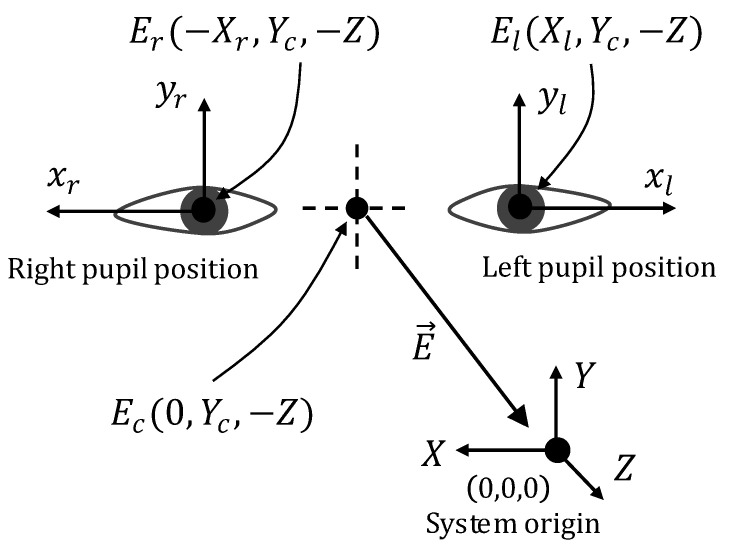
Pupil positions.

When a human watches a 3D POI (*D*), as shown in Figure 1, a vector of eye gaze ($\vec{E}$) is theoretically drawn from the center ($E_{c}$) of both the left and right pupils to the POI (*D*). The eye tracker [30] that was used in this paper normally provides the positions of the left and right pupils. These positions are then used in order to calculate the center ($E_{c}$) of both pupils, and a 2D POG (*A*), which is a eye gaze point on the virtual board, is used with the mentioned center point ($E_{c}$) to create the vector of the eye gaze ($\vec{E}$). In this paper, the system origin is initially established at the center of the eye tracker, and the positions of the extracted left and right pupils are located at the coordinates $E_{r}\left(-X_{r},Y_{c},-Z\right)$ and $E_{l}\left(X_{l},Y_{c},-Z\right)$, respectively, as shown in Figure 3.

**Figure 4:**
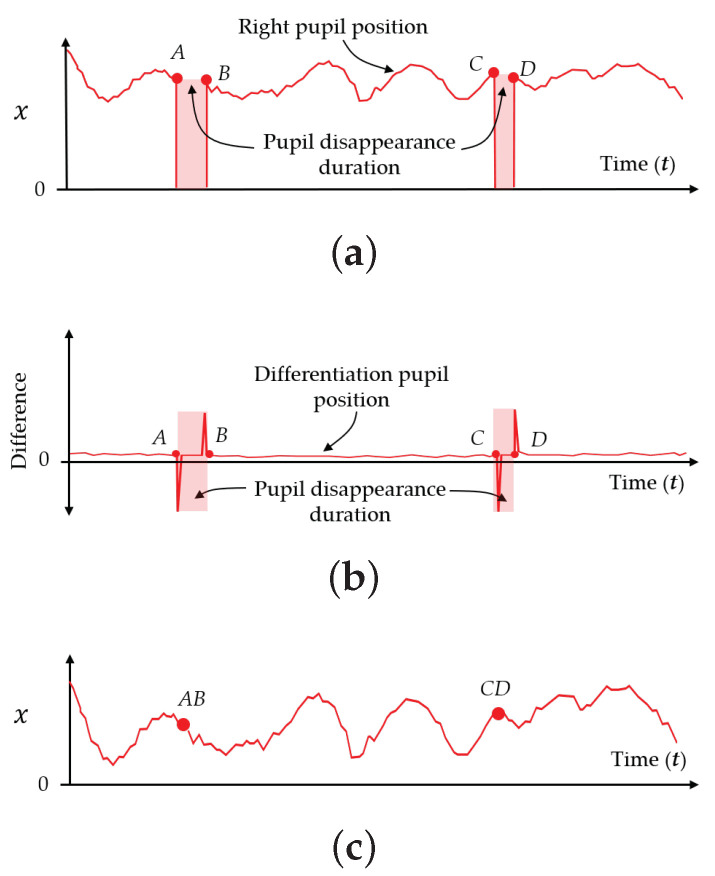
Deletion of pupil disappearance duration: (**a**) Pupil disappearance duration, (**b**) Differentiation of pupil position, and (**c**) Modified of pupil position.

Humans normally blink their eyes [42] and cause the eye tracker to sometimes lose track of the pupil positions, as seen in the observation results of the left and right eyes over time. The duration of this tracking loss is called the pupil disappearance duration in this paper. In addition to the constant saccades of human eyes, it is found that the pupil positions sometimes disappear in the data series of pupil positions in the time domain. Therefore, the saccades and blinks of the human eye cause vibrations and outstanding peaks, respectively, as shown in Figure 4a. W. Pichitwong and K. Chamnongthai [33] take the average to determine the representative position of the pupil and, therefore, errors occur due to pupil disappearance. If we take derivatives between consecutive time frames, the differentiation results detect peaks that represent the starting and ending points of a blink or pupil disappearance duration, as shown by (AB and CD) duration in Figure 4b. These pupil disappearance durations are detected and ignored in the method proposed in this paper, and the original signal representing the pupil position becomes an analog signal including only the vibration of saccades, as shown in Figure 4c. Therefore, the average position of the signal is improved as compared with that in the conventional method [33].

### 3.3. Definition of Hand Pointing Shapes

**Figure 5:**
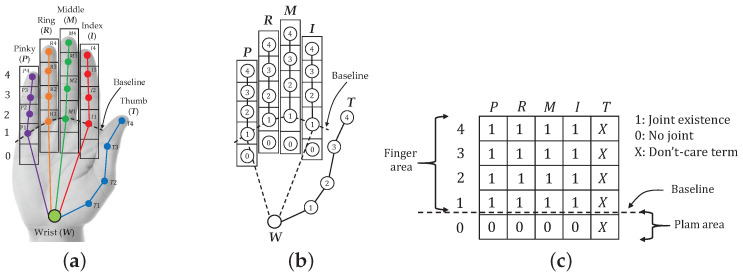
Definition of hand and template: (**a**) Finger joints and skeleton, (**b**) Template, and (**c**) Matrix.

A hand shape can basically be represented based on finger joints as a feature [20], a gesture of hand pointing can be established as a hand shape pattern in a still image, and humans naturally pause their hand posture in a pointing pattern position for a period of time during pointing [43]. This paper analyzes the hand pointing mode, considering two parts as necessary conditions: static finger gestures and the hand posture during pointing. The hand pointing mode is used to switch the mode of the intention estimation. Suppose that a fingertip and four joints of each finger can be detected by a sensor [44], as shown in Figure 5a, which are T1−4, I1−4, M1−4, R1−4, and P1−4. Physiologically, the first joints of all fingers (T1, I1, M1, R1, and P1) are fixed without any movement, and a line passing those joints can be assumed to be a baseline for considering the movement of other finger joints.

Regarding the first condition of static finger gestures, while the thumb is free as the don’t-care term, the index finger absolutely needs to be a straight line in the pointing gesture, and the pinky, ring, and middle fingers have to be bent inward. Although these three bent fingers are slightly varied in vertical direction in reality, the bent fingers that represent hand pointing can be concluded in some patterns, which will be discussed in detail in the next subsection. If we set up rectangles to cover all of the finger joints and tips, with the tips and joints being located in the center of the rectangle, a template consisting of rectangular cells can be obtained, as shown in Figure 5b. In some cases in which the fingers are bent inward lower than the baseline, other cells (P0, R0, M0, and I0) are established to cover some possible patterns of the fingertips of bent fingers. Finally, if we informatively assign the logical “1” as a hand and “0” as no hand in the cells of the matrix, the pattern of the hand pointing template is obtained, as shown in Figure 5c.

### 3.4. Hand Pointing Angle and Coordinate

**Figure 6:**
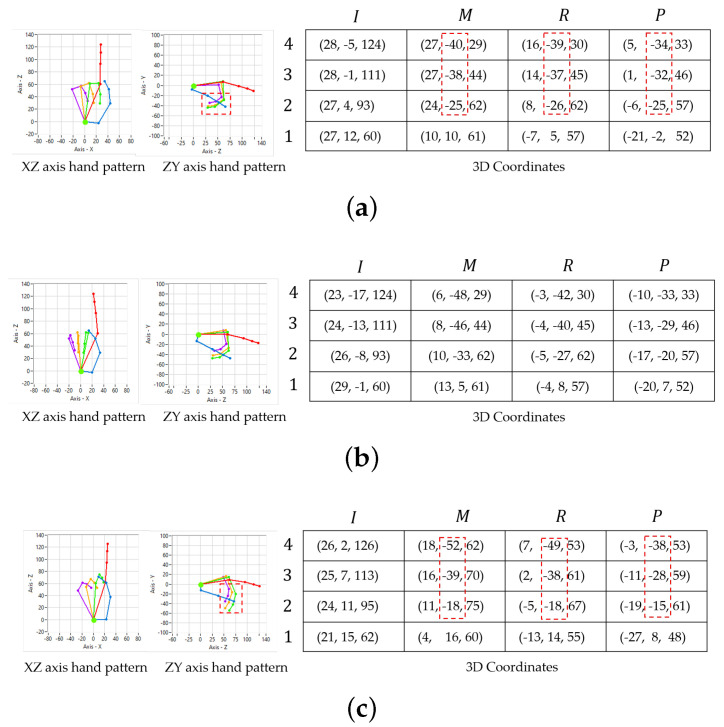

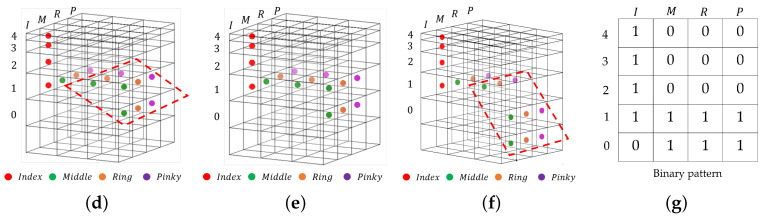
Comparison of a hand pointing pattern in different postures: (**a**) Hand pointing in origin posture with coordinates, (**b**) Hand pointing in rotated posture, (**c**) Hand pointing with slight changes, ring, and pinky finger, (**d**) 3D binary pattern of (**a**), (**e**) 3D binary pattern of (**b**), (**f**) 3D binary pattern of (**c**), and (**g**) Two-dimensional (2D) binary patterns of (**a**,**c**)

Originally, hand and finger are sensed by a Leap Motion sensor in the unit of hand pointing detection, and the coordinates of 15 finger joints and five fingertips are obtained. These coordinates are input data for the determination of hand pointing. Naturally, hand and fingers are freely moved and located in any postures, and coordinate patterns of hand pointing would be varied according to the hand postures. Although hand pointing patterns are fixed in a finite number, the number of hand pointing patterns may be increased according to the hand postures and this may disable the pattern matching. The coordinates of finger joints and fingertips would be first rotated to the original angle before the pattern matching process in order to solve the problem. For instance, coordinates of hand pointing and rotated hand pointing, as shown in Figure 6a,b, respectively, are converted into a 3D binary pattern, as shown in Figure 6d,e, respectively. Obviously, the 3D binary patterns of both coordinate examples are different due to the rotated posture. Thus, the coordinates of finger joints and fingertips should be rotated to the origin posture in the first step. Moreover, when a user performs a hand pointing gesture many times, the hand pointing gestures may exactly be different, due to slight changes of bending pinky, ring, and middle fingers, as shown in Figure 6a,c. Apparently, these slight changes may not affect the meaning of hand pointing, but 3D binary patterns are differentiated, as shown by the parts that are surrounded by red dash lines presented in Figure 6d,f. However, it can be observed that coordinates in *Y* axis of middle, ring, and pinky fingers are largely different when compared with the ones in *X* and *Z* axes, as shown by red dash-line rectangles in shown Figure 6a,c. If only coordinates of *X* and *Z* axes are converted into a 2D binary pattern, then both of coordinates, as shown in Figure 6a,c, would become the same pattern, as shown in Figure 6g. This means that 2D information of finger joints and fingertips can absorb the slight changes of those pinky, ring, and middle fingers, and many 3D binary patterns of a hand pointing gesture can be unified into a 2D binary pattern. Therefore, the 3D coordinates of finger joints and fingertips should be projected into 2D ones before finding a binary pattern.

### 3.5. Hand Pointing Pattern

**Figure 7:**
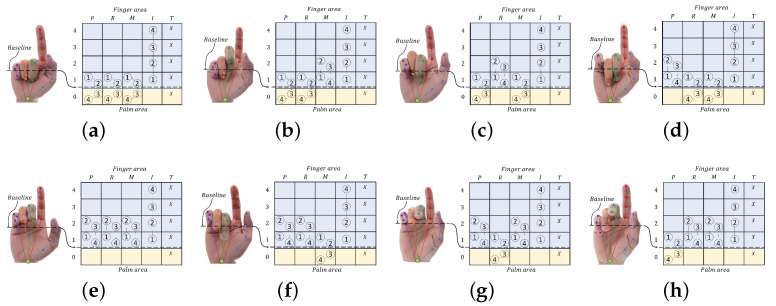
Hand pointing patterns: (**a**) Pattern $PT_{1}$, (**b**) Pattern $PT_{2}$, (**c**) Pattern $PT_{3}$, (**d**) Pattern $PT_{4}$, (**e**) Pattern $PT_{5}$, (**f**) Pattern $PT_{6}$, (**g**) Pattern $PT_{7}$, and (**h**) Pattern $PT_{8}$.

With our basic concept of hand pointing described above, the gestures of all five fingers should be digitally analyzed into one of the possible patterns for hand pointing. These possible hand patterns are used as templates to classify hand pointing. While the thumb is logically regarded as a don’t-care term, the index finger then has to be a straight line as the first condition, and the remaining three fingers, the middle, ring, and pinky, fingers physically have to be bent inward as the second condition. In bending the middle, ring, and pinky fingers, these fingers are not always in the same positions, even for the same person. This means that there exists more than one possible pattern of bending for these three fingers when a hand intentionally points. By using a sensor, such as the Leap Motion sensor [45], which can digitally sense the positions of a fingertip and three finger joints of each finger, the patterns of a fingertip and three finger joints of these three fingers in a bent position after 3D-to-2D projection can be grouped into eight patterns in terms of probability and physiology, as shown in Figure 7, in which all of the joints of these three fingers are located below the middle of the index finger.

This means that all four joins of the middle, ring, and pinky fingers have to be physically located below joint “2” of the index finger. When the index finger (*I*) behaves as a straight line and all four joints are located in four individual cells in the vertical direction, all three fingers (*M*, *R*, and *P*) are located based on *I*1 and the cell below I1, as shown in $PT_{1}$, and each of the three fingers is shifted to one upper cell from left to right, as shown in $PT_{2}$, $PT_{3}$, and $PT_{4}$. Upon shifting all of the bent fingers up by one cell to I2, $PT_{5}$ to $PT_{8}$ show the hand pointing with three bent fingers located below I2. All four joints of the three fingers (*M*, *R*, and *P*) are located at the same level as I2 and I1, as shown in $PT_{5}$. Each of the three fingers, “middle, ring, or pinky”, in $PT_{5}$ is shifted down one cell to form the other three patterns of $PT_{6}$, $PT_{7}$, and $PT_{8}$, respectively. These are the possible patterns of hand pointing that are used to recognize and confirm the hand pointing shape in this paper.

**Figure 8:**
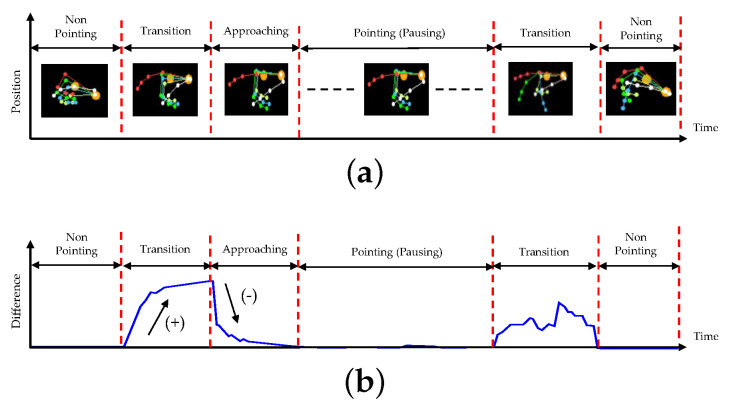
Pause duration and transition: (**a**) A sequence of finger joint form, (**b**) Differences of consecutive frames.

On the other hand, the hand posture must pause for a time in the second condition of the hand pointing mode. If we analyze the movement of finger joints in a video sequence, then the finger joints in a pointing pattern and other patterns are shown in the video sequence, as shown in the example presented in Figure 8a. If we simply differentiate consecutive frames, the results may indicate differences during non-pointing, transition, and approaching durations and no difference during pause durations, as shown in Figure 8b. Therefore, this paper proposes identifying the candidates of the hand pointing mode by detecting the first frame of the pause duration, performing pattern matching with the first frame and each of the eight trained patterns, and confirming that the hand pointing vector is located in the SOI. When the hand pointing mode is confirmed, the closest point between the eye gaze and hand pointing vectors is then obtained as the POI.

## 4. System Overview

**Figure 9:**
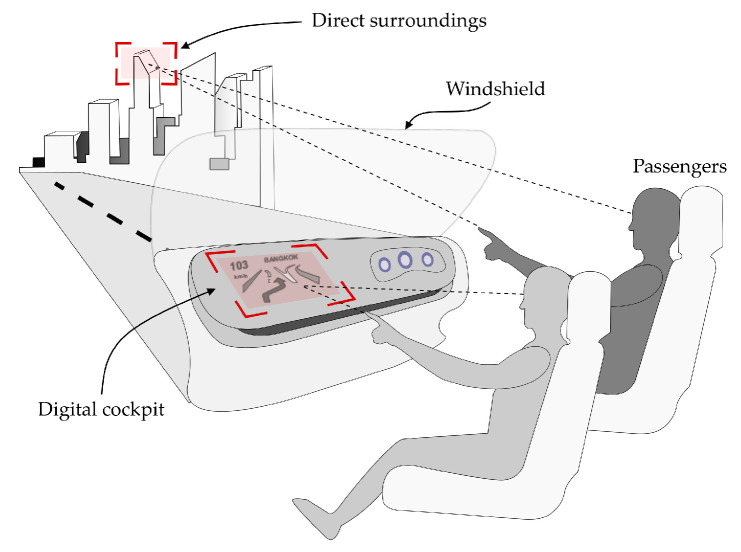
Scenario of a human-machine interface in a vehicle.

While facilities in living and working environment, such as home, hospital, factory, and so on, tend to be more smart, convenient, and friendly for users, vehicles, such as cars, airplanes, and ships, are becoming increasingly advanced due to their related technologies. Currently, it is obvious that our living and working environments will be changed to be conveniently controlled by users in remote places, and cars will become auto-driving, without requiring a driver, and will also be much more capable of communicating with and performing services for passengers. One of the keys to communicate and interface with users is to know the intentions of all of the users. Some users may not be free to move to the control switches in the living and working environments, and some passengers may not sit in the front of the passenger space. Therefore, they will not be able to touch a touchscreen or push buttons on the front console; thus, 3D virtual screens may become effective for interfacing with passengers, as shown by the scenario depicted in Figure 9. Our proposed system is described in two parts, which are the hardware and software units, below.

### 4.1. Hardware Unit

**Figure 10:**
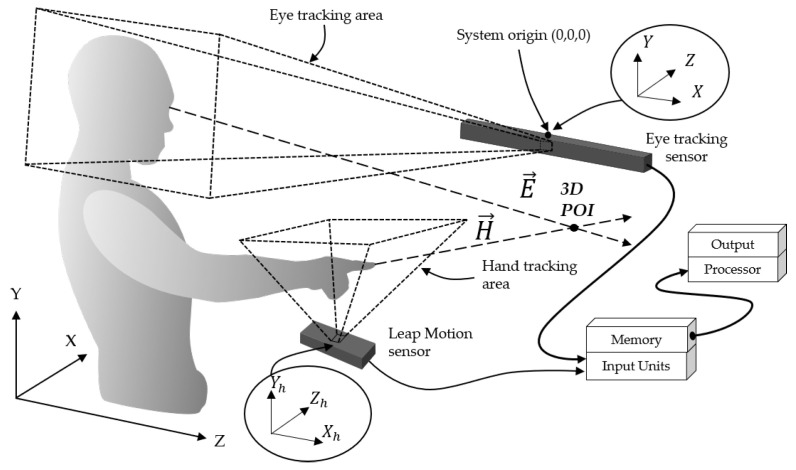
Overview system of multimodal of eye gaze and hand pointing.

In our system overview, the hardware system consists of an eye tracker [30], the Leap Motion sensor [45], memory, processor, and input/output units, as shown in Figure 10. The Leap Motion sensor, which can sense hand gestures, is installed in front of the user. In this paper, the range covered by the Leap Motion sensor is called the hand tracking area. In addition, an eye tracker is set up in front of the user, so that it can detect the user’s eyes, and the range that is covered by the eye tracker is called the eye tracking area in this paper.

The origins of the coordinates of the eye tracker (*X*, *Y*, *Z*) and Leap Motion sensor ($X_{h}$, $Y_{h}$, $Z_{h}$) are located in the center of each sensor and, in practice, the coordinates of the hand tracking area are initially calibrated to the coordinates of the eye tracking sensor. In testing, the eye gaze ($\vec{E}$) and hand pointing ($\vec{H}$) directions are detected by the eye tracking sensor and Leap Motion sensor, respectively. The 3D POI is geometrically found at the crossing point between the hand-pointing and eye gaze directions.

### 4.2. Software Unit

**Figure 11:**
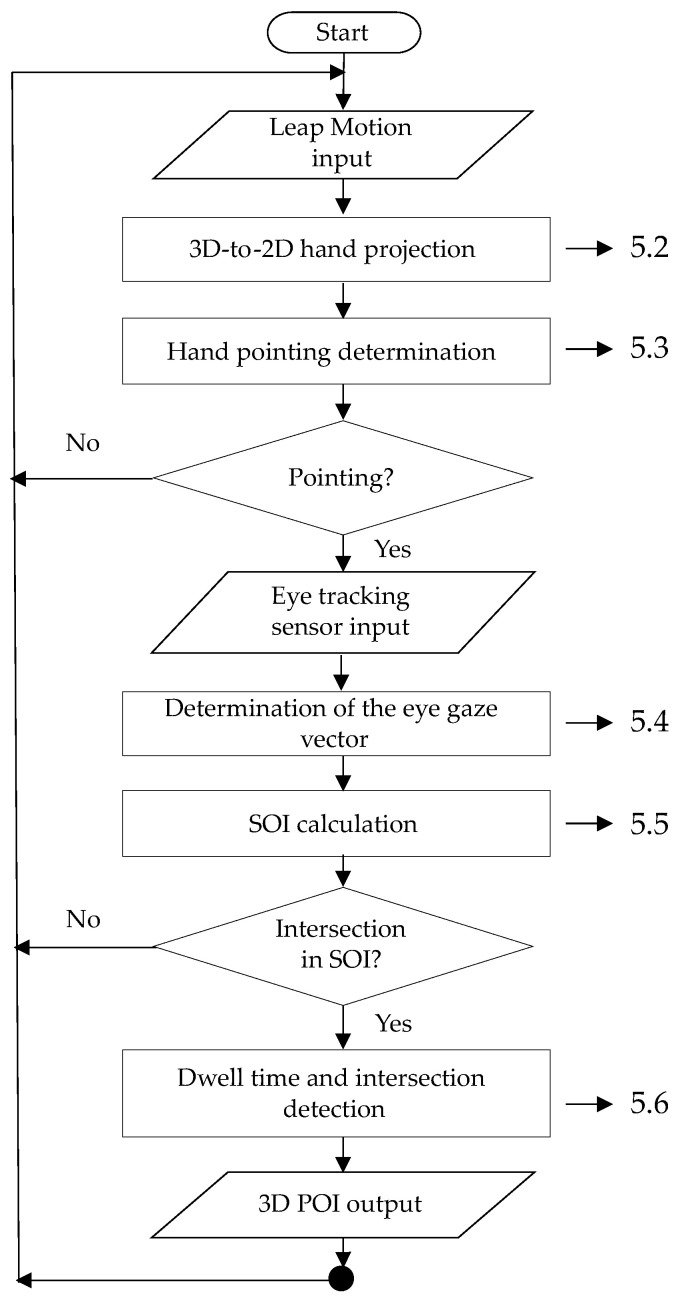
Flowchart of proposed method.

Based on the established hardware, our software unit starts with the Leap Motion input process, which senses the finger joint positions with the Leap Motion sensor, as shown in Figure 11. The unit then projects the 3D finger joint-position data to 2D data in the 3D-to-2D hand projection process and detects hand pointing in the hand-pointing determination process. Section 5.2 and Section 5.3, respectively, describe these processes. In the case in which the hand is not determined to be in pointing mode, the system does not process further and it goes back to the Leap Motion input process. If the system decides that the hand is in pointing mode, the eye gaze is input from the eye tracker in the eye tracking sensor input process, the eye gaze vector is determined in the process of determination of the eye gaze vector, and the SOI is calculated in the SOI calculation process, which are processes introduced in Section 5.4 and Section 5.5, respectively. At this time, if the vector of hand pointing intersects the SOI, then the system is confirmed to be in the pointing mode. The system then finds the closest 3D point between the vectors of eye gaze and hand pointing, which is assumed to be the POI. The details of the closest-point calculation are reported in Section 5.6. Otherwise, the system returns to start the loop again, with new finger joint positions being obtained by the Leap Motion sensor.

## 5. Proposed Methods

The calibration and processes of the flowchart shown in Figure 11 are realized, as follows, in order to implement our basic concept of the proposed 3D POI determination method using multimodal fusion of the eye gaze and hand pointing for a 3D display.

### 5.1. Calibration

**Figure 12:**
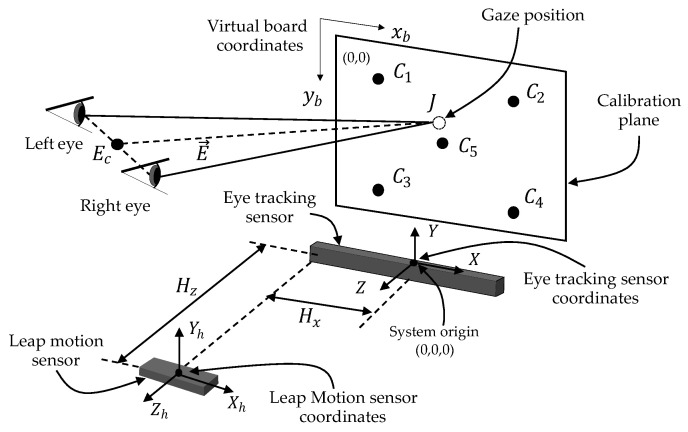
System calibration.

There are three coordinate systems: those of the eye tracker (*X*, *Y*, *Z*), hand tracker ($X_{h}$, $Y_{h}$, $Z_{h}$), and virtual board ($x_{b}$, $y_{b}$), as shown in Figure 12. In practice, users have to calibrate these three coordinate systems to find connection the virtual board and hand tracker coordinates with the coordinates of the eye tracking sensor in advance. The eye tracking sensor is assumed to obtain the coordinates of the left and right pupil centers and the 2D eye gaze position on the virtual board. In the calibration, a board with some points that represent the priors of the measured 3D position ($C_{1}$–$C_{5}$) on the eye tracker coordinates is temporarily installed, and the 2D coordinates of those five points on the board [46] ($C_{1}$–$C_{5}$) are the outputs from the eye tracking sensors when an examiner looks at those five points. The conversion between the 2D virtual board and 3D eye tracking sensor coordinates is performed with the following equations:

(6) $$R_{x}=\frac{N}{2}\left(\left|\frac{C_{1}\left(y\right)-C_{2}\left(y\right)}{C_{1}\left(x\right)-C_{2}\left(x\right)}\right|+\left|\frac{C_{3}\left(y\right)-C_{4}\left(y\right)}{C_{3}\left(x\right)-C_{4}\left(x\right)}\right|\right)$$

(7) $$R_{y}=\frac{N}{2}\left(\left|\frac{C_{1}\left(y\right)-C_{3}\left(y\right)}{C_{1}\left(x\right)-C_{3}\left(x\right)}\right|+\left|\frac{C_{2}\left(y\right)-C_{4}\left(y\right)}{C_{2}\left(x\right)-C_{4}\left(x\right)}\right|\right)$$

where, *N* is a fixed distant value with real-world units (millimeters), and the resulting value *R* is the ratio of the converted eye gaze position in terms of pixels to millimeters.

(8) $$J\left(x,y\right)=R_{x}\left(G\left(x\right)-C_{5}\left(x\right)\right),R_{y}\left(G\left(y\right)-C_{5}\left(y\right)\right)$$

where, *G* is the position (*x*, *y*) of the eye gaze in units of pixels. The resulting value *J* is the real-world value according to the ratio *R* and it is offset by $C_{5}$.

Subsequently, the distances between the origins of the hand tracker and eye tracker ($H_{z}$ and $H_{x}$) are manually measured, so that the transformation between the hand tracker and eye tracker coordinates can be obtained. During the testing state, the board that is used in calibrations is removed, and this is called the “virtual board” in this paper.

**Figure 13:**
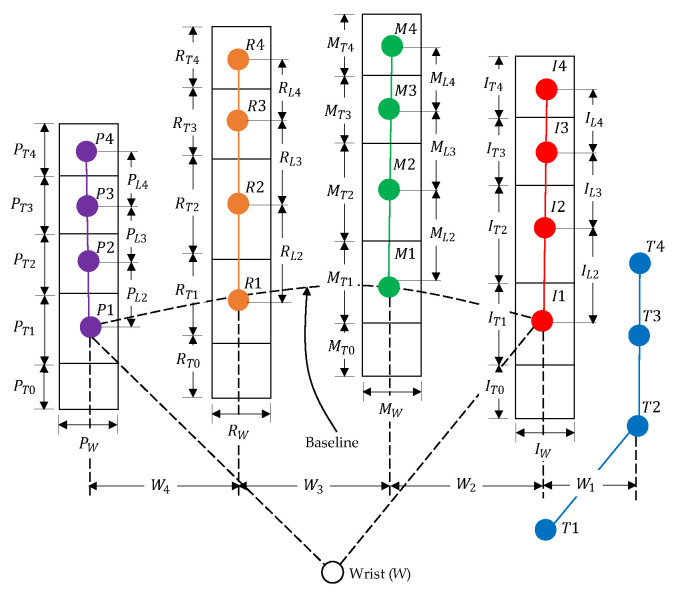
Template range determination.

As another necessary calibration, the hand pointing template has to be established in advance. Because the hand and finger sizes of each user are different, the cell sizes have to be measured by a hand sensor, and the hand-pointing template has to be determined. The ranges between neighboring joints and tips ($I_{L2-4}$, $M_{L2-4}$, $R_{L2-4}$, and $P_{L2-4}$) are measured according to coordinates from a sensor, and rectangular cells are assumed to be established to cover all fingertips and joints, as shown in Figure 13. The sizes of the template cells ($I_{T1-4}$, $M_{T1-4}$, $R_{T1-4}$, and $P_{T1-4}$) can be simply obtained by the following equations:

(9) $$F_{T4}=\left(F_{4}+\frac{F_{L4}}{2}\right)-\left(F_{3}+\frac{F_{L4}}{2}\right)$$

(10) $$F_{T3}=\left(F_{3}+\frac{F_{L4}}{2}\right)-\left(F_{2}+\frac{F_{L3}}{2}\right)$$

(11) $$F_{T2}=\left(F_{2}+\frac{F_{L3}}{2}\right)-\left(F_{1}+\frac{F_{L2}}{2}\right)$$

(12) $$F_{T1}=\left(F_{3}+\frac{F_{L2}}{2}\right)-\left(F_{1}+\frac{F_{L2}}{2}\right)$$

(13) $$F_{T0}=F_{T1}$$

where, F={I,M,R,P}.

On the other hand, the ranges between neighboring finger joints on the baseline ($W_{1}$–$W_{4}$) are measured according to the coordinates from the hand motion, and the width of the template cells (Iw, Mw, Rw, and Pw) can be calculated by the following equations:

(14) $$I_{W}=\left(I_{1}+\frac{W_{1}}{2}\right)-\left(M_{1}+\frac{W_{2}}{2}\right)$$

(15) $$M_{W}=\left(M_{1}+\frac{W_{2}}{2}\right)-\left(R_{1}+\frac{W_{3}}{2}\right)$$

(16) $$R_{W}=\left(R_{1}+\frac{W_{3}}{2}\right)-\left(P_{1}+\frac{W_{4}}{2}\right)$$

(17) $$P_{W}=\left(P_{1}+\frac{W_{4}}{2}\right)-W_{4}$$

Finally, the bottom cells of those four fingers (I0, M0, R0, and P0) are originally for some bent fingers that possibly cross over the baseline, such that their sizes are assumed to be the same as those on the baseline (I1, M1, R1, and P1).

### 5.2. 3D-To-2D Hand Projection

In the viewpoint of the Leap Motion sensor that is installed below the hand, finger joints are sometimes considered to be occluded in the palm or other fingers. This is likely to cause errors in finger joint extraction. The view angle of the hand should be facing the sensor at the appropriate angle to obtain the finger joint positions and convert them to a template. Therefore, it is required to rotate [47] the hand (*X*, *Y*, *Z*) at the wrist to the desired angle with the following equation:

(18) $$\left[\begin{array}{c} X^{\prime }\\ Y^{\prime }\\ Z^{\prime } \end{array}\right]=R_{Z}\left(\psi \right)R_{Y}\left(\theta \right)R_{X}\left(\phi \right)\left[\begin{array}{c} X\\ Y\\ Z \end{array}\right]$$

**Figure 14:**
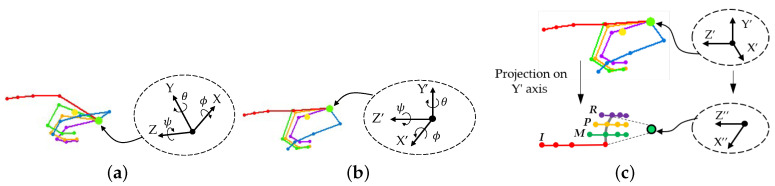
3D-to-2D hand projection: (**a**) 3D hand skeleton detection, (**b**) Rotation, and (**c**) 2D projection.

The finger joints of the five fingers of the hand, as shown in Figure 14a, are then rotated to the desired angle, as shown in Figure 14b. Now, the hand angle is ready to detect all of the finger joints, assuming no occlusion, and this is a good angle at which to project [48] the 3D position ($X^{\prime }$, $Y^{\prime }$, $Z^{\prime }$) onto the 2D ($X^{"}$, $Z^{"}$) position by the following Equation (19), as shown in Figure 14c.

(19) $$\left[\begin{array}{c} X^{"}\\ Z^{"} \end{array}\right]=\left[\begin{array}{ccc} J_{X} & 0 & 0\\ 0 & 0 & J_{Z} \end{array}\right]\left[\begin{array}{c} X^{\prime }\\ Y^{\prime }\\ Z^{\prime } \end{array}\right]$$

Algorithm 1 depicts the algorithm of 3D-to-2D hand projection.
**Algorithm 1** Algorithm for 3D-to-2D hand projection.**Input:** 3D finger joint positions *J***Input:** 3D wrist position *W*  1: **compute**
J=J−W  2: **for**
*i* each column **do**  3:  **for**
*j* each joint **do**  4:   **compute** rotation of $J\left[i\right]\left[j\right]\left(X^{\prime },Y^{\prime },Z^{\prime }\right)$  5:   **compute** projection of $J\left[i\right]\left[j\right]\left(X^{\prime },Y^{\prime },Z^{\prime }\right)⟶J\left[i\right]\left[j\right]\left(X^{\prime \prime },Z^{\prime \prime }\right)$  6:  **end for**  7: **end for**


### 5.3. Hand Pointing Determination

**Figure 15:**
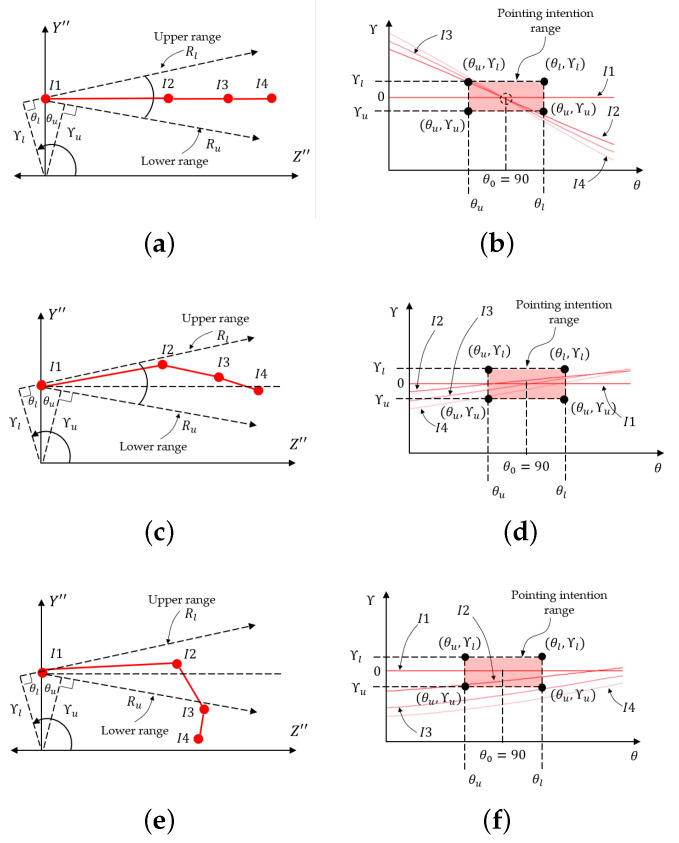
Index fingers with Hough transform: (**a**) Straight index finger, (**b**) Straight index finger in Hough domain, (**c**) Bending index finger considered as straight, (**d**) Bending index finger in Hough domain, (**e**) Straight index finger with failed determination, and (**f**) Failed straight index finger in Hough domain.

The 2D positions ($X^{\prime \prime }$, $Z^{\prime \prime }$) of five joints have been projected from 3D finger joint positions by the previous process of 3D-to-2D hand projection. In order to determine the 2D projected hand shape, which is either hand pointing or non-pointing, two conditions are used in this paper: (1) the index finger has to be a straight line and (2) the middle, ring, and pinky fingers have to be bent inward [34]. In the first step, the four joints of the index (I1, I2, I3, and I4) are geometrically checked to confirm a straight line. The Hough transform [49] is used in this paper to check whether the three line parts segments (the lines drawn between I1 and I2, I2 and I3, and I3 and I4) are either the same straight lines or different straight lines. As shown in Figure 15a, the three line segments (lines drawn between I1 and I2, I2 and I3, and I3 and I4) that are represented in red are converted by the Hough transform to become three points in the Hough coordinates (θ, γ), as shown in Figure 15b. In the ideal case, in which all three lines are on the same straight line, all of the straight lines are converted to the same point, which is located at point H(90,γ).

(20) $$H=\left(\theta_{u}<\theta_{p}⩽\theta_{l}\right)\wedge \left(\gamma_{u}<\gamma_{p}⩽\gamma_{l}\right)$$

In practical cases, the index finger might be slightly bent and, physiologically, the human index finger can bend up to 30 degrees at most [50], so this paper proposes training the maximum acceptable angle range, which is called the pointing intention range, from some samples in advance. The trained pointing intention range must be within the limit of 30 degrees for the upper and lower angles above and below from the target line. In the case in which the human intends to point to a 3D POI target, the three lines mentioned are expected to be geometrically located between two upper and lower acceptable-error range vectors, $R_{u}$ and $R_{l}$, which define the so-called pointing intention range, as shown in Figure 15c.

In order to convert this range into Hough coordinates, the pointing intention range converted by the following Equation (20) is used; it determines the pink rectangle shown in Figure 15d. If the human has no intention to point to a 3D POI, not all of the vectors associated with the index finger may be geometrically located in the pointing intention range, as shown in Figure 15e, and at least one of the points representing the three straight lines mentioned has to be outside the rectangle of the pointing intention range, as shown in Figure 15f. Algorithm 2 conducts the straight-line check for the index finger.
**Algorithm 2** Algorithm for straight index finger detection.**Input:** range of θ as $\theta_{u}$ and $\theta_{l}$
**Input:** range of γ as $\gamma_{u}$ and $\gamma_{l}$
**Input:** index finger joints as I=[I1,I2,I3,I4]
  1: **for**
*i* each joint of index finger **do**  2:  **for**
*j* = 0 to 180 **do**  3:   **compute**
I[i](Z,Y) as the Hough transform of each *j* degree $⟶H_{t}\left(\gamma ,\theta \right)$  4:  **end for**  5:  **calculate** intersection of $H_{t}\left(\gamma ,\theta \right)$ and $\gamma_{0}$$⟶(\gamma_{p},\theta_{p})$  6:  **if**
$\theta_{u}<\theta_{p}⩽\theta_{l}$ and $\gamma_{u}<\gamma_{p}⩽\gamma_{l}$
**then**  7:   **set** index finger straight  8:  **else**  9:   **set** index finger not straight10:  **end if**11: **end for**

**Figure 16:**
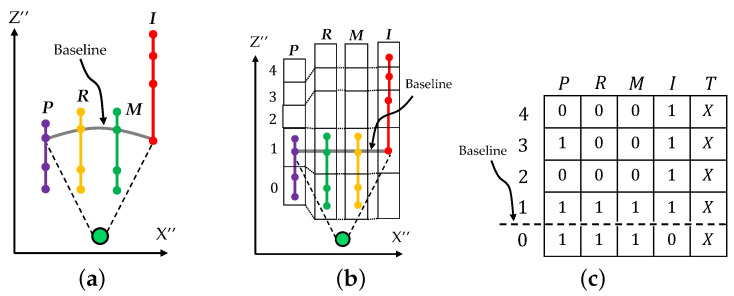
Template matching: (**a**) Hand skeleton projection, (**b**) Template matching and (**c**) Matrix of binary pattern.

If the index finger is confirmed to be a straight line as the first necessary condition, then the hand pointing template is applied to the 2D projected hand. Suppose that the coordinates of the fingertips and joints are obtained as input data, as shown in Figure 16a. The data are matched with the template for hand pointing determination. Practically, the template is separated into a one-dimensional (1D) matrix for each finger, and each matrix is applied based on the baseline of the finger, as shown in Figure 16b.

All of the cells are checked for the existence of tips and joints, with “1” and “0” used for existence and non-existence, respectively, to create a matrix, as shown in Figure 16c. The matrix is then used to perform pattern matching with the eight trained templates of the hand pointing. Template matching for hand pointing determination (HP) is logically performed with the eight templates (PT1–PT8), according to the following equations:

(21) HP=T∧(PT1∨PT2∨⋯PT8)

(22) $$HP=\left\{\begin{array}{cc} 0; & \mathrm{Non}\;\mathrm{matching}\\ 1; & \mathrm{Matching} \end{array}\right.$$

If one of the eight templates is matched with the hand shape, the output of the equation logically results in “1”, which means that the hand is pointing, as shown in the equation above. In the cases in which the hand shape is not matched with any of the eight templates, the logical result is “0”, which indicates non-pointing. Algorithm 3 concludes the pattern matching of hand pointing.
**Algorithm 3** Algorithm for pattern matching.**Input:**PT1,PT2,PT3,PT4,PT5,PT6,PT7,PT8**Input:** hand pattern as *T* at current frame  1: **for**
*i* each pattern of templates **do**  2:  **calculate**
HP=T∧(PT1∨PT2∨..PT8)  3:  **if**
HP = 1 **then**  4:   **set** pattern match  5:  **else**  6:   **set** pattern does not match  7:  **end if**  8: **end for**

**Figure 17:**
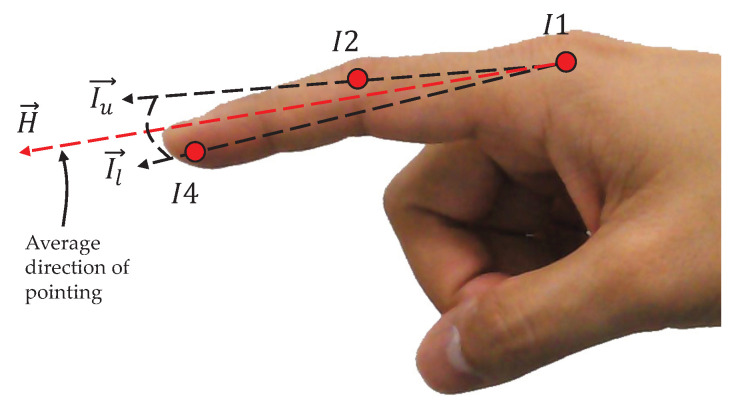
Hand-pointing direction estimation.

In order to estimate the hand pointing direction in the final process of the subroutine, the index is physiologically defined to be a straight line on the straight-line drawn between I1 and I4 [51], as shown in Figure 17. In practice, the index finger, which is intentionally pointed in a straight line directly toward a target, may not always form a real straight line, but makes slight angles at the joints. Therefore, this paper establishes an acceptable range, called the pointing intention range, for absorbing small acceptable errors in the index gesture and determines the index pointing vector according to the average direction between the two vectors formed by I1 and I2 and by I1 and I4, as shown by the dashed line presented in Figure 17.

The index is assumed to be intentionally pointing to a target if the index pointing vector is located within the pointing intention range. The pointing intention range is initially established by training on samples of pointing human hands, and this paper practically uses 20 degrees for the upper and lower angles above and below as the range.

(23) $$\vec{H}=\vec{I_{u}}+\vec{I_{l}}$$

### 5.4. Determination of the Eye Gaze Vector

**Figure 18:**
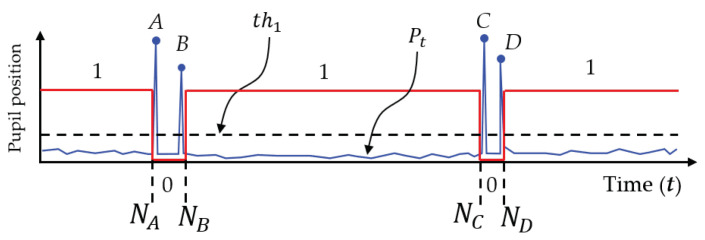
Thresholding for pupil-disappearance duration determination.

Based on the concepts shown in Section 3.2, this paper proposes determining the pupil disappearance duration (AB and CD) and deleting it to correct the analog signal representing the pupil positions ($P_{t}$). In implementing this concept, the analog signal representing the pupil positions, as shown in Figure 4a, is calculated according to the threshold value ($th_{1}$) to detect the pupil disappearance duration as shown in Figure 18, that is defined by the following equation:

(24) $$th_{1}=\frac{F}{N}\sum_{t=1}^{N}\left|P_{t+1}-P_{t}\right|$$

where *F* and *N* represent the parameters of the threshold value and the number of time frames, respectively. In thresholding, the differentiation of consecutive time frames ($D_{p}$), which is performed by Equation (25), is binarized in order to find the starting and ending points of the pupil disappearance durations.

(25) $$D_{p}\left[t\right]=\left\{\begin{array}{cc} 0; & D_{p}<th_{1}\\ 1; & D_{p}⩾th_{1} \end{array}\right.$$

where, t=1,2,⋯,N. The differentiation time frames with a logical “1” are regarded as the starting and ending points of the pupil disappearance duration, and the signal components during this duration should be deleted for correction. The corrected signal components that represent the pupil positions are found as the average pupil positions. Subsequently, left and right representative pupil positions are both used to find the center point ($E_{c}$), and the center point is used with the 2D POG obtained from the eye tracking sensor to draw a straight line, which is regarded as the vector of the eye gaze.

### 5.5. Soi Calculation

Human line-of-sight rays may move within an approximate range of 5–10 degrees due to saccades, according to the research using works in psychological experiments [39,40]. Therefore, this paper sets the maximum cornea movement range to cover covering saccades, which is called the SOI, as shown in Figure 2b. When the eye tracker senses both pupil centers, the light ray making a right angle with the straight-line between both eyes and passing through a point on the virtual board may be used to determine the visual field based on the aforementioned maximum cornea movement.

(26) $$\vec{V_{u}}=r\cos\left(\theta_{e}\right),r\sin\left(\theta_{e}\right)$$

(27) $$\vec{V_{l}}=r\cos\left(\theta_{e}\right),r\sin\left(\theta_{e}\right)$$

### 5.6. Dwell Time and Intersection Detection

**Figure 19:**
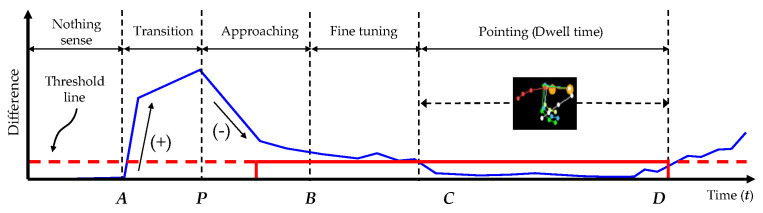
Thresholding for finding pause as pointing duration.

When pointing at a target, a user normally moves the current hand shape to the hand pointing shape, and then pauses for a time, as shown by the example presented in Figure 19.

At first, the sensor may sense nothing, and the detected hand velocity may be zero, as shown in the beginning of the graph in Figure 19, when the hand disappears in the hand tracking area. As a hand sensor, the Leap Motion sensor senses a hand whenever a hand appears in the hand tracking area. The hand velocity may sharply increase when a hand appears in the hand tracking area (*A*), and the hand takes the shape of pointing; this is called the transforming state. When the hand shape transforms, and has almost reached the hand pointing shape (*P*), the hand velocity rapidly decreases; this is called the approaching state. Subsequenyly, the hand enters a mode of adjusting the hand shape (*B*), which is called the fine-tuning state, and the hand velocity fluctuates slightly until it reaches a nearly zero level. The hand in the pointing shape may be paused for a while during the dwell time. The best time to find the target of hand pointing is the starting point of the dwell time (*C*), as observed.

To detect the dwell time, we should start by finding the first non-zero point (*A*) and then perform smoothing to delete local spikes in the signal, as shown in Algorithm 4. Subsequently, the slopes of all parts of the signal are calculated; positive and negative slopes representing the transforming and approaching states, respectively, are found; and the end of the approaching state or beginning of the fine-tuning state (*B*) is detected. To find the starting point of the dwell time or the end of the fine-tuning state, ideally, it is possible to find the first zero point. However, there is noise in reality, and the hand velocity in the dwell time is not absolutely zero or even close to zero. Practically, the noise should be suppressed by thresholding, and the first zero point then becomes the starting point of the dwell time. The thresholding value should be initially set by rate comparison to the average of the signal from *B* to *D* in pretesting.
**Algorithm 4** Algorithm for dwell time detection.**Input:** data frame from Leap Motion as *L*  1: set i=0  2: **while**
$V_{i}\neq 0$
**do**  3:  i=i+1  4: **end while**⟶ A  5: **calculate** smoothing (i→n)  6: **calculate** slope finding (-) and (+) ⟶B  7: **compute** thresholding ⟶ suppress noise  8: **compute** find zero

**Figure 20:**
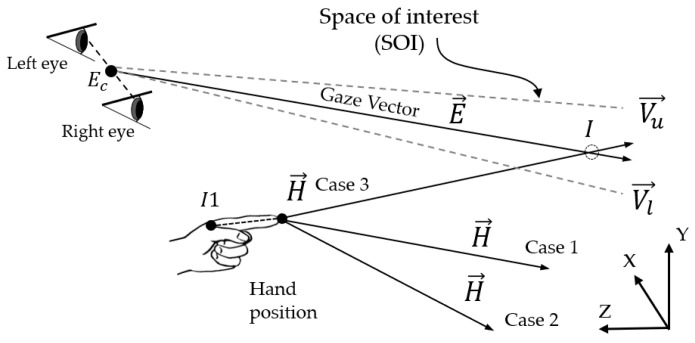
Crossing point determination using closest point principle.

A line representing hand pointing can logically be categorized into one of three cases, Cases 1–3, as shown in Figure 20. In Case 1, the straight-line representing the hand pointing direction intersects the visual field at a point behind zero, while the intersected point is located in front of the zero point in Case 3. Case 2 shows the hand pointing direction, which is a ray parallel with the edge of the SOI. When Case 3, which is a suspected candidate for the pointing intention, is detected, there needs be a pause, called the “dwell time”, according to a study of human intention [52,53]. The pause time should be initially measured by pretesting with examiners, and it is thereafter used to check the dwell time for intention determination.

When an intersection is detected during the dwell time, the closest point [54,55] between the hand pointing vector and eye gaze is calculated (28)–(32).

(28) $$\vec{P}=I_{1}-E_{c}$$

(29) $$m=\frac{\left(\left(\vec{E}\cdot \vec{H}\right)\left(\vec{P}\cdot \vec{E}\right)\right)-\left(\left(\vec{P}\cdot \vec{H}\right)\left(\vec{E}\cdot \vec{E}\right)\right)}{\left(\left(\vec{H}\cdot \vec{H}\right)\left(\vec{E}\cdot \vec{E}\right)\right)-\left(\left(\vec{E}\cdot \vec{H}\right)\left(\vec{E}\cdot \vec{H}\right)\right)}$$

(30) $$R_{a}=I_{1}+m\vec{H}$$

(31) $$R_{b}=E_{c}+\vec{E}\left(\frac{\left(\left(\vec{P}\cdot \vec{E}\right)\right)+m\left(\vec{E}\cdot \vec{H}\right)}{\left(\vec{E}\cdot \vec{E}\right)}\right)$$

(32) $$I=\frac{R_{a}+R_{b}}{2}$$

where $E_{c}$ is the middle point between the left and right pupil positions.

### 5.7. Limitation of Proposed Method

In the implementation of the system based on the basic concept, users should realize some limitations to prevent errors. Those limitations are explained, as follows.

#### 5.7.1. Head Fixation

The proposed POI determination method basically uses a light ray vector making a right angle with the straight line between both eyes and passing through a fixation point on the virtual board to assist determination of POI by finding a crossing point with the vector of hand pointing. The head angle is regarded to sensitively influence the right angle of the mentioned right ray vector, and it may induce errors in POI detection. Users should raise awareness of the angle of the participant head, and fix the head in calibration, training, and testing processes.

#### 5.7.2. Hand Size Measurement and Hand Pointing Training

Normally, every person has different sizes of hands and fingers. The hand template and patterns are calibrated and determined based on the sizes of the participant hand and fingers, as shown in Figure 5 and Figure 6. This means that a hand template and hand patterns belonging to a person cannot be used for others. Users should be cautious of this matter and try to set the hand template and patterns for each person. In addition, the shape of the index finger during hand pointing also depends on a person in which all of the participants may perform different gestures of the index finger. In practice, these should be trained for each person and then tested based on the trained data of the same person to avoid errors.

#### 5.7.3. Poi Occlusion against Eye Gaze

**Figure 21:**
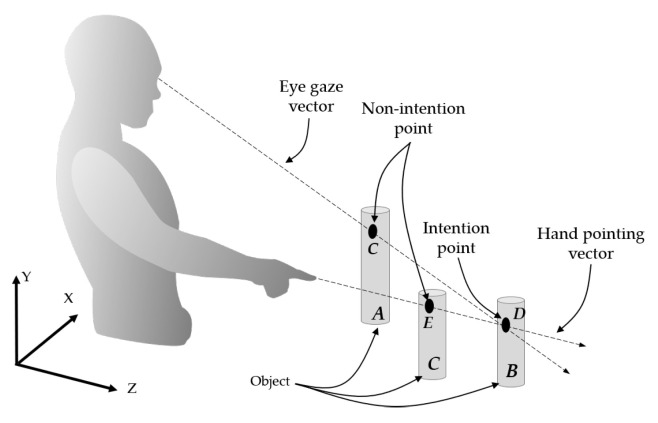
Intended point detection when eye gaze is obstructed by an object.

Our proposed POI determination method basically deals with a POI in a 3D space. Sometimes POI is located behind other objects. If an object (*C*) obstructs the vector of hand pointing, this method can find the POI, as shown in Figure 21. However, in the case that another object (*A*) occludes the eye gaze of the participant, the proposed method actually cannot find the POI. This kind of occlusion on the eye gaze becomes the limitation of our proposed method.

#### 5.7.4. Available Processing Time

According to psychologist experiments [53], a person may pause the hand pointing gesture within approximately 350–600 milliseconds. The POI determination system should complete all of the processes within the mentioned duration to prevent errors of the hand pointing vector and POI position. This is exactly a condition for the system design and development, and the time condition is regarded as another limitation.

#### 5.7.5. Size of SOI

Human eyes naturally saccade in different range. The eye saccade range is used in this proposed method in order to determine the SOI based on the maximum distance of the POI. In our experiments, the average saccade range that is based on statistical data of many people is used to set the angle of the SOI cone. Precisely, saccade should be measured personally, and the measured angle of saccade range at the maximum distance of POI should be exactly used in the testing process.

## 6. Experiments and Results

In order to evaluate the performance of the proposed 3D POI determination using multimodal fusion of the hand pointing and eye gaze, experiments with 10 participants, five males and five females, were performed based on the case study specifications that are shown in Table 1.

**Figure 22:**
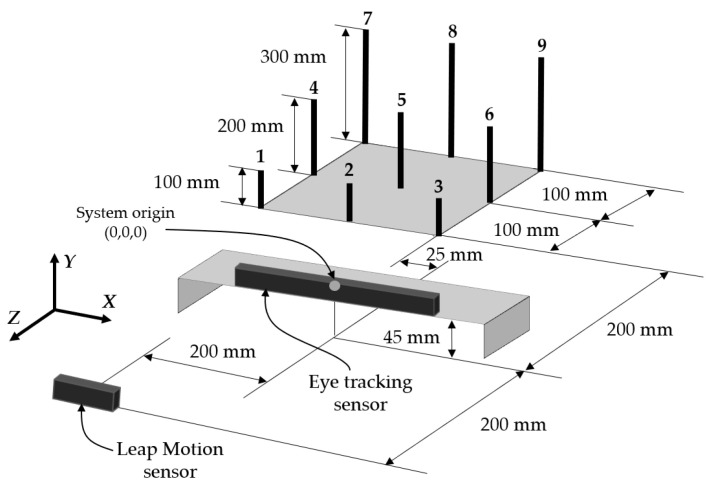
Experimental equipment model.

**Figure 23:**
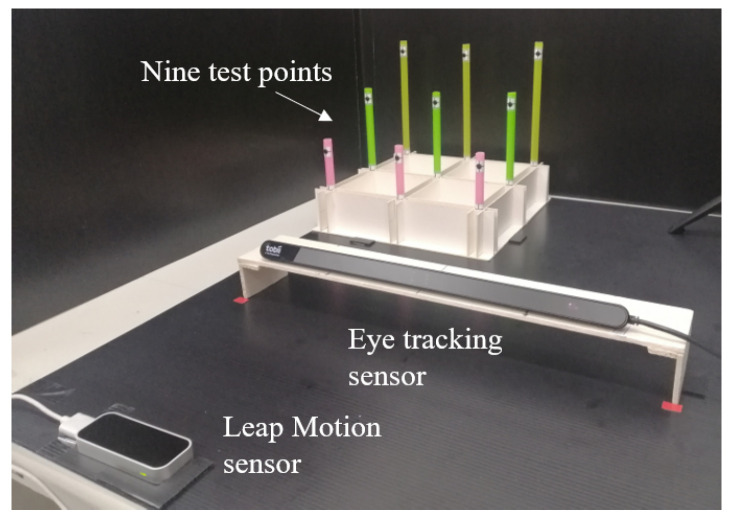
A photograph of an experimental equipment.

An eye tracking sensor and hand tracking sensor that detect both pupils and all joint positions of the hand, respectively, were used as sensors, and a currently popular computer, operating system, and software were selected as the basic experimental system. The experimental equipment was conceptually designed in the layout that is shown in Figure 22. In the equipment, nine poles were installed on the left-hand side or right-hand side of the examiner, depending on whether they were left or right-handed, as shown in Figure 23.

**Figure 24:**
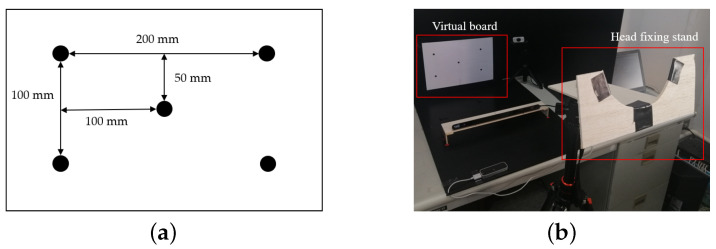
Virtual board: (**a**) Layout of points on a virtual board, (**b**) Photograph of a virtual board with a head fixing stand.

**Figure 25:**
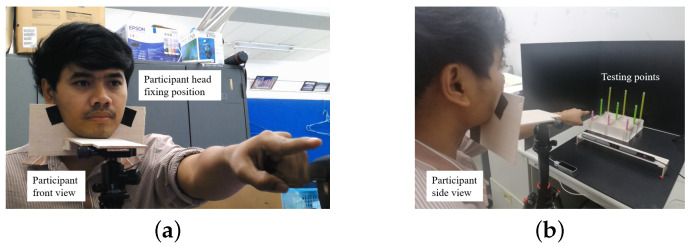
Photographs of head position fixed in the experiments: (**a**) Front view, and (**b**) Side view.

The system origin was set at the center of the eye tracking sensor, so that the measurement results of the eye tracker, which are the 2D coordinates of the virtual board and the 3D coordinates of the hand joints, were calibrated to the same origin. Calibrations of the eye tracker were initially performed by a virtual board that was made from a piece of paper. Figure 24a,b show the layout of the virtual board and its photo. In the calibrations for converting points on the virtual board (2D) to points on the 3D coordinates, in which the origin is located in the center of the eye tracker, a person who is trained puts his/her chin on the fixed head stand to stabilize the head for several seconds, as shown in Figure 25a.

The obtained coordinates (*X*, *Y*) of the five points on the virtual board and the measured 3D coordinates (*X*, *Y*, *Z*) of those five points on the board are used to fix the required parameters for the perspective transform, as shown in Figure 24b. These parameters and the perspective transform were applied in the calibrations that are mentioned in Section 5.1, and experiments on the multimodal fusion of eye and hand tracking were then performed, as shown in Figure 25b. Before testing, all of the participants were advised and trained the usage of experimental instruments until their ability was confirmed.The testing processes were performed in the same manner as those in the training state. Since hand size and hand pointing shape may differ among participants, each participant was calibrated and tested based on the individual trained data. Figure 25b shows examples of the processing of the hand-pointing pattern determination, index-finger straight detection, and eye gaze positions on nine poles in the experiments.

**Figure 26:**
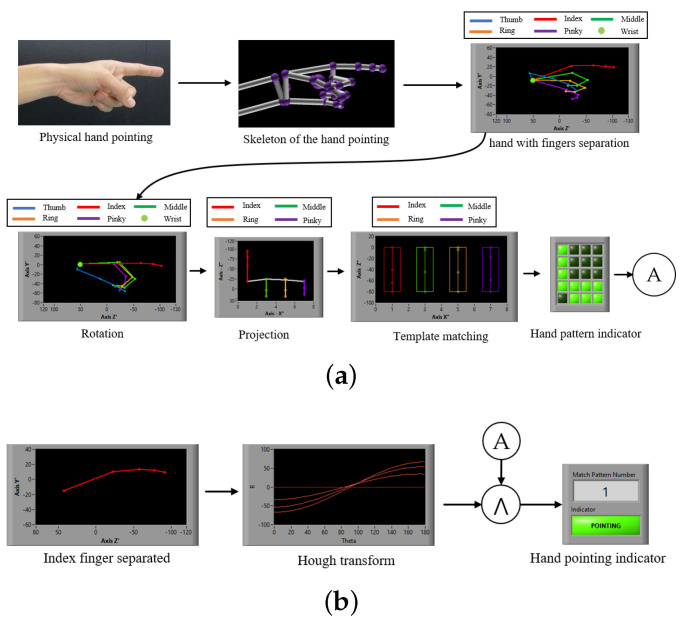
Example of process of hand pointing determination: (**a**) Hand pointing determination and (**b**) Index finger straight detection.

**Figure 27:**
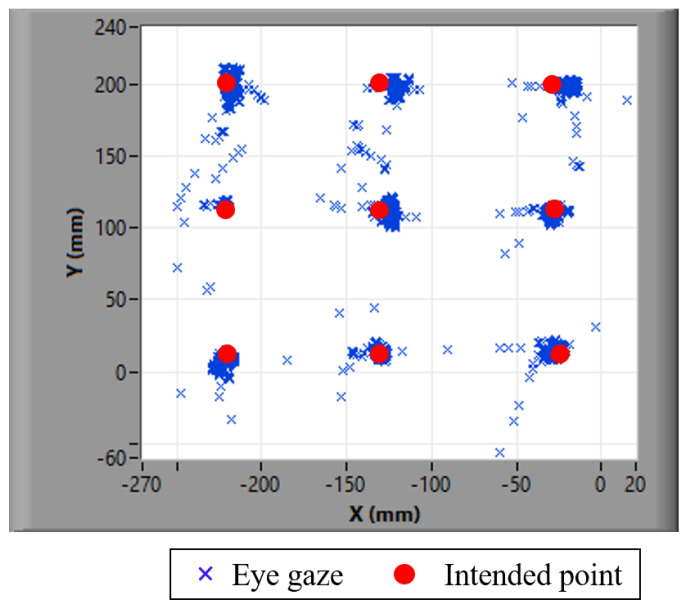
Trajectories of eye gaze at the intended points of nine poles.

Figure 26a,b show an example of the experimental results of hand-pointing patterning matching and straightness detection of the index finger, respectively. Figure 27 shows the experimental results of eye gaze detection against nine poles.

The experimental results of the hand pointing mode are shown for a number of different testing samples presented in Table 2. Table 3 shows the probability of hand-pointing pattern matching in each pattern obtained from our experiments.

An evaluation of the proposed method for 3D coordination measurement was performed, and Table 4 shows the results. These measurement accuracies were compared with the results of the conventional methods in 2D, as shown in Table 5.

## 7. Discussion

In order to express an intended 3D POI to others, humans normally point to the POI by hand pointing, or hand pointing with eye gaze. Hand pointing has been studied as the best priority to reveal intention on a POI based on visual estimation. Although methods of POG detection using eye gaze proved to be excellent ways to detect a point of gaze, the detected points of gaze or POGs are not necessary to be POI. To determine intention of human based on gestures, hand pointing is absolutely like a switch to trigger the intention. The authors of this paper have improved the decision of hand pointing vectors as compared with the conventional methods [22,23] for POI detection on 2D screen, as shown by Euclidean distance errors presented in Table 5.

In the case of expressing an intended a POI in 3D space, another vector is required to find a crossing point, which can be either hand pointing with eye gaze or hand pointing with head direction [56]. Originally, it was proven that eye gaze is more precise than head direction, so that this paper proposed selecting a multimodal combination of hand pointing with eye gaze to find 3D POIs. The experimental results shown in Table 4 proved that performance of the combination of hand pointing and eye gaze is workable for 3D POI determination. Moreover, the proposed method can deal not only 3D, but also 2D as accuracy comparison with conventional methods for 2D POI, as shown in Table 5.

In future vehicles that are autonomously navigated without human drivers, passengers may feel free to work or enjoy activities in the passenger area, and a virtual human-machine interface that applies 3D POIs may play a larger role as a smart infrastructure in the cabin. Moreover, some counter services in the future may apply 3D POIs to decrease the number of human workers. These environments are examples in which 3D POIs will become increasingly essential, and users in these environments may not normally want to move their head and face to express intentions, because it is not a typical human method. Hand pointing is basically accepted as a good and accurate way for humans to show intention, but finding another straight line to exactly determine a POI on the line of hand pointing is required. This paper proposes the utilization of the eye gaze, which can be detected as another straight-line for POI determination in 3D space. In practice, there are many cases in which the two straight lines never cross in the 3D space due to human eye saccades and device errors and, thus, a point needs to be determined as the crossing point.

Therefore, this paper proposes the determination of the crossing point according to the closest distance between the two straight lines. The experimental results presented in Table 4 and Table 5 show that our proposed method achieved an accuracy greater than 90% within a POI distance of one meter.

**Figure 28:**
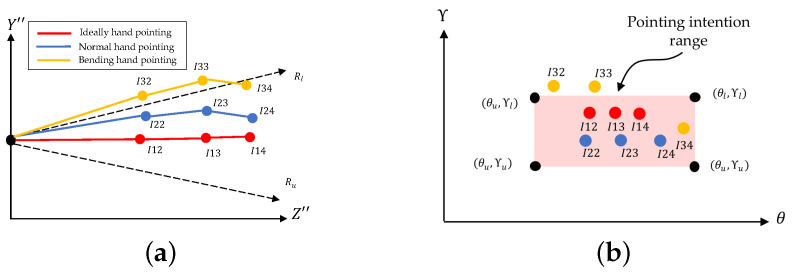
Hand error during pointing mode: (**a**) Index finger over range during pointing mode and (**b**) Error caused from over range during pointing mode.

In the error analysis, the errors of 3D POI determination are divided into two groups: errors in hand pointing determination representing intention and errors in multimodal-fusion-based 3D POI determination. In the first group of hand-pointing determination errors, it is divided into two factors that are straight index finger detection and template matching. As shown in Table 2, the accuracy of the first and second factors reach 95.53% and 100%, respectively. The errors of the straight index finger detection can be considered and analyzed in finger bending as the cause. The yellow index finger comes up over the thresholding range of the dash line, as shown by an example in Figure 28a. It is also obvious in the Hough domain that the yellow points representing straight index fingers, which are over the range, are allocated outside the pointing intention range, as shown in Figure 28b. Although this is actually close to the border of straight index finger detection, it is counted as out of the range based on the threshold. The thresholding value has been set to ensure the intention of hand pointing so that some located close to the border may not be picked up. In order to solve this problem, the training of hand pointing should be trained well enough to ensure the pointing ability.

**Figure 29:**
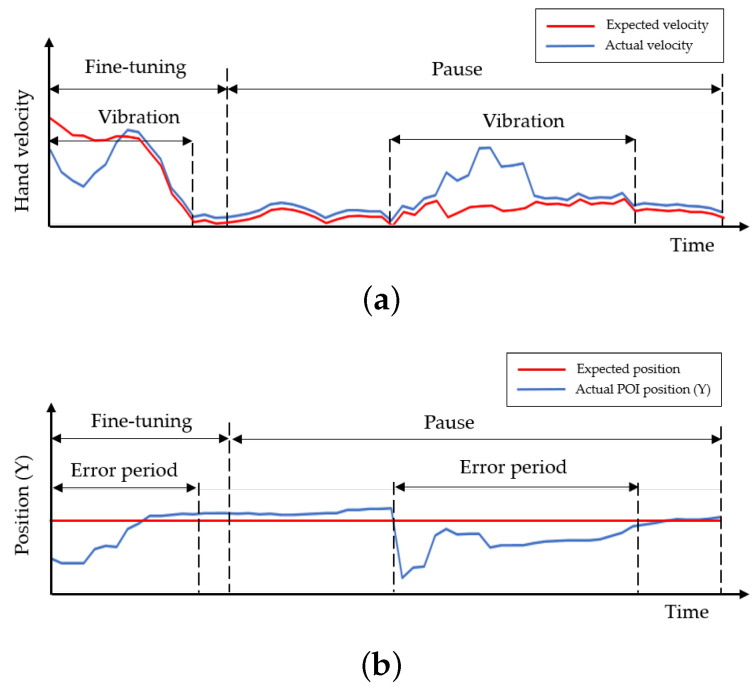
Error of straight index finger determination caused by vibration during hand pointing: (**a**) Index finger vibration, and (**b**) Index finger position.

Originally, the index finger should be paused during the pausing period, but it vibrated slightly, as shown in Figure 29a. This caused an error in the position of the index finger, as shown in Figure 29b. In the Hough coordinates, some points were located outside of the rectangle representing the pointing intention range. In fact, the pointing intention range was initially determined based on the average data of training samples, and it is regarded as the average among a group of people. Sometimes, this is not suitable for all users. The pointing intention range should be trained individually for each user tso solve this problem. In the second cause of index finger vibration, the human index finger sometimes vibrates slightly, and its range normally varies, depending on the person. Our paper solves this problem by taking the average position as the pointing vector, which is not guaranteed to represent the real pointing vector. The pointing characteristics that are regarded as depending on a person may be reconsidered, and users may individually retrain the system in the training state.

**Figure 30:**
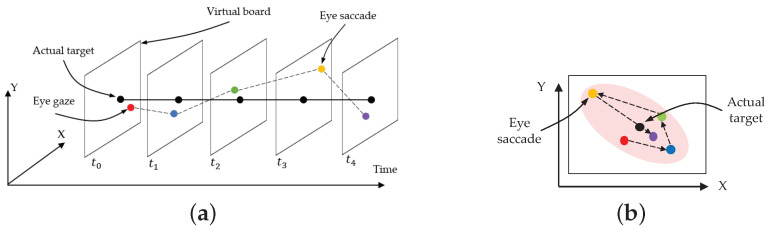
Error caused by saccade: (**a**) Eye gazes in time domain, and (**b**) Eye gazes in Hough domain.

For the second error group of multimodal-fusion-based 3D POI determination according to the experimental results presented in Table 4, the eye gaze is ideally concentrated on a target of 3D POG. In reality, eye saccades always occur, and they cause errors in eye gaze tracking, as shown in Figure 30b. In the time domain, the eye gaze vibrates around the POI, as shown in Figure 30a, and these become errors that are caused by saccades within a range, as shown in Figure 30b. This should be considered statistically in solving this problem in future work. In addition, the hand pattern matching performed well in the experiments, as shown by the results presented in Table 2, because all of the participants were trained appropriately before the experiments. Users are recommended to be trained before testing to avoid errors in hand pattern matching in real applications.

Finally, the proposed method of 3D POI detection while using a multimodal fusion of hand pointing and eye gaze was shown to be able to find an acceptable 3D POI and it may often contribute to the virtual world in the future. As a trade-off in development of 3D POI detection system, users may obtain an excellent function of 3D POI detection, while users have to invest costs of some instruments such as eye tracker, Leap Motion sensor, processor, memory, and so on. Although these devices currently become much lower cost with more excellent functions, users should consider the total investment cost in the view point of worth paying. Moreover, excellent functions, which can help elderly, patient, and disabled people, may be traded off with complexity of the developed system. This method obviously utilizes two sensors, which are an eye tracker and a Leap Motion sensor, in implementation. Although the system has good accuracy and enables 3D POI determination, the investment cost and complexity of the system are regarded as trade-offs of the proposed method.

## 8. Conclusions

Finding a 3D POI, which plays an important role in the development of smart human-machine interfaces in virtual worlds, requires reliability in terms of both accuracy and real-time operation in practice. In some special applications, such as in a cockpit, users are not allowed to move their head, face, or body due to safety reasons; therefore, the fusion of the eye gaze and hand pointing is an effective and human-friendly method of determining a 3D POI. Because the eye gaze has recently been shown to be reliably detected in determining a POG on a 2D screen, the straight-line that is drawn from the pupil to the POG on the screen can be extended to a 3D POI, although 3D POI determination still requires another straight line that crosses the straight-line extended in space. Hand pointing, which is considered to be a natural way for humans to indicate a 3D POI, can be used to address this issue. However, if the hand moves freely in the 3D space, then a hand pointing mode showing human intention should be defined to avoid intention errors. This paper proposes determining the hand pointing mode by first determining the hand pause duration and then comparing hand gestures that have straight index fingers and bent middle, ring, and pinky fingers. Subsequently, the hand pointing vector drawn along the straight index finger is confirmed to be within the SOI, and the 3D POI is finally determined based on the crossing point or closest distance between the two vectors of hand pointing and the eye gaze. Experiments that were performed with 10 participants show that the proposed method can measure 3D POIs located at a distance of one meter with an average distance error of approximately 5.25%.

## Figures and Tables

**Table 1:** System configuration and experimental setup.

Devices/Software/Participants	Specification
Eye tracking sensor	Tobii model 4C [30] Operating distance: 50–95 cm Track box dimensions: 40 × 30 cm at 29.5${}^{"}$/75 cm
Hand tracking sensor	Leap Motion sensor [45] 150-degree field of view 60 frames per second
Computer	Intel Core i3—7130U 2.7 GHz RAM 4 GB
Display	Resolution 1920 × 1080 pixels Screen size 445 × 250 mm
Operating system	Windows 10 64 bit
Software	LabVIEW 2018 Student 32 bit Tobii EyeX SDK Leap Motion SDK
Participants	5 males and 5 females

**Table 2:** Hand pointing determination accuracy.

Testing	Accuracy (%)
Straight index finger detection	95.53
Template matching	100

**Table 3:** Appearance probability of hand patterns.

Pattern Number	Matched (%)
1	50.84
2	3.46
3	1.59
4	0
5	26.20
6	0.54
7	0
8	17.34

**Table 4:** Euclidean distance error of the proposed method in 3D spaces.

Testing Point	Testing Distance (cm)	X (cm)	Y (cm)	Z (cm)	XYZ (cm)	Error (%)
1	75.0	1.03	1.40	2.01	2.85	3.80
2	75.0	1.02	1.82	2.82	3.75	5.00
3	75.0	0.59	1.86	3.05	3.90	5.20
4	85.0	2.24	1.19	3.09	4.57	5.38
5	85.0	2.02	1.06	3.91	4.87	5.73
6	85.0	1.29	1.69	3.17	4.28	5.04
7	95.0	2.18	1.68	3.76	5.00	5.26
8	95.0	1.12	2.02	4.66	5.51	5.80
9	95.0	1.44	2.18	4.60	5.76	6.06
Average		1.44	1.66	3.45	4.50	5.25
SD.		0.58	0.37	0.86	0.91	0.65

**Table 5:** Comparison of POI detection errors between conventional and proposed methods in 2D and 3D spaces.

Testing Distance (cm)	Euclidean Distance Error			
	2D		2D	3D
	Index Finger (Tip) [22]	Index Finger (Tip and Carpals) [23]	Hand Pointing Vector (Proposed Method)	Hand Pointing and Eye Gaze (Proposed Method)
8.0	∼0.68 cm (8.47%)	-	-	-
16.0	∼2.38 cm (14.87%)	-	-	-
50.0	-	1.65 cm (3.30%) by $\left[1.46^{\circ },1.20^{\circ }\right]$	2.23 cm (4.46%)	2.81 cm (5.62%)
75.0	-	-	2.68 cm (3.57%)	4.67 cm (4.67%)
80.0	-	3.45 cm (4.31%) by $\left[1.97^{\circ },1.49^{\circ }\right]$	2.14 cm (2.67%)	4.16 cm (5.20%)
85.0	-	-	2.94 cm (3.46%)	5.38 cm (5.38%)
95.0	-	-	3.54 cm (3.72%)	5.71 cm (5.71%)
110.0	-	5.84 cm (5.31%) by $\left[2.45^{\circ },1.80^{\circ }\right]$	-	-

## Data Availability

Not applicable.

## Abbreviations

The following abbreviations are used in this manuscript:

POI	Point of Intention
2D POI	Two-Dimensional Point of Intention
3D POI	Three-Dimensional Point of Intention
POG	Point of Gaze
2D POG	Two-Dimensional Point of Gaze
3D POG	Three-Dimensional Point of Gaze
SOI	Space of interest
AOI	Area of Interest
